# Structure-guided engineering of prototype foamy virus Env identifies key residues for heparan sulfate binding and enhances transduction efficiency

**DOI:** 10.3389/fbioe.2026.1716928

**Published:** 2026-01-26

**Authors:** Hee-Seung Shin, Soo-Yeon Cho, Yujin Kwon, Seong-Mook Jung, Eun Sang Seo, Young Min Son, Eui Tae Kim, Doyoun Kim, Kyoung-Dong Kim

**Affiliations:** 1 Department of Systems Biotechnology, Chung-Ang University, Anseong, Republic of Korea; 2 Therapeutics & Biotechnology Division, Rare Disease Therapeutics Research Center, Korea Research Institute of Chemical Technology (KRICT), Daejeon, Republic of Korea; 3 Department of Medicinal Chemistry and Pharmacy, Korea University of Science and Technology (UST), Daejeon, Republic of Korea; 4 Department of Microbiology and Immunology, Jeju National University College of Medicine, Jeju, Republic of Korea; 5 Department of Biomedicine & Drug Development, Jeju National University, Jeju, Republic of Korea; 6 School of Pharmacy, Sungkyunkwan University, Suwon, Republic of Korea

**Keywords:** env engineering, gene delivery, heparan sulfate, prototype foamy virus, receptor-binding domain, transduction efficiency

## Abstract

**Objective:**

Prototype foamy virus (PFV) is an attractive gene delivery platform due to its large cargo capacity and favorable safety profile; however, the structural basis of its interaction with heparan sulfate (HS), a critical attachment factor for viral entry, remains undefined. The objective of this study was to identify the structural determinants of HS recognition within PFV Env and to evaluate whether rational, structure-guided engineering could enhance viral entry and gene transfer efficiency.

**Methods:**

We applied a structure-guided engineering strategy combining *in silico* structural modeling, molecular docking, and systematic mutagenesis of the PFV Env receptor-binding domain (RBD), targeted residue substitutions, and combinatorial mutations spanning the upper domain (UD) and lower domain (LD) were generated and evaluated using quantitative cell-based transduction assays. In addition, Tet-On-inducible Env-expressing stable producer cell lines were established to provide a reproducible platform for functional validation.

**Results:**

Alanine substitutions at R298, R440, and E446 in the UD abolished infectivity, confirming their essential roles in HS-mediated attachment. In contrast, selective substitutions at adjacent positions, Q296R and G403F in the UD, and E232N, I330F, and I334F in the LD, enhanced transduction efficiency by up to 1.32-fold relative to the wild type. Combinatorial variants integrating beneficial UD and LD mutations exhibited synergistic effects, achieving a transduction efficiency of 68.9%, corresponding to a 1.55-fold increase over the wild type (44.4%). Interspecies domain replacement with simian foamy virus Env reduced infectivity, underscoring the context-specific nature of PFV-HS interactions. In the inducible stable cell system, the LD var6 mutant achieved 8.6% transduction compared to 4.4% for the wild type, representing up to a 1.95-fold increase.

**Discussion:**

These findings define the structural determinants of HS recognition in PFV Env and demonstrate that residue-level, structure-guided engineering can enhance PFV transduction efficiency. This study provides experimentally validated insight into PFV Env-HS interactions and establishes a rational framework for further optimization of PFV-based gene delivery technologies.

## 1 Introduction

Gene therapy has emerged as a promising approach for treating a wide range of inherited and acquired diseases, with the efficacy and safety of this approach depending on the gene delivery vehicle (Naldini, 2015; Zu and Gao, 2021; Lundstrom, 2023). Viral vectors like adenoviruses (AdVs), adeno-associated viruses (AAV), lentiviruses (LV), and gamma-retroviruses (γRVs), have been developed and applied in preclinical and clinical settings (Ginn et al., 2024). However, each vector platform has its limitations. For example, AdV and AAV vectors can induce potent immune responses, whereas LV and γRV systems pose risks of insertional mutagenesis and oncogenesis due to random genomic integration (Colella et al., 2018; Shirley et al., 2020; Banou et al., 2024). In addition, these vectors have a limited gene insertion capacity and low transduction efficiency in certain cell types, which continues to limit their broader applicability (Hendrie and Russell, 2005; Liu et al., 2020; Pirona et al., 2020). These limitations highlight the pressing need to develop safer and more versatile gene delivery systems that combine high efficiency, low immunogenicity, and minimal genotoxicity.

Recent advances in viral vector engineering have shown that modifications of capsid or Env (Envelope glycoprotein) proteins can enhance cell-type specificity, transduction efficiency, and immune evasion. In AAV systems, targeted capsid mutations, particularly tyrosine-to-phenylalanine substitutions at surface-exposed residues (e.g., Y444, Y500, and Y730), improve intracellular trafficking and transgene expression by reducing ubiquitination and proteasome-mediated degradation (Zhong et al., 2008; Petrs-Silva et al., 2009; Petrs-Silva et al., 2011). In addition to single-site changes, domain shuffling has generated highly infectious chimeric capsids, such as AAV-DJ (Grimm et al., 2008), whereas rationally designed hybrids, such as AAV2.5, have improved muscle tropism and reduced antigenicity (Rabinowitz et al., 2002). AAV-based gene therapies have since advanced to clinical testing in patients with muscular dystrophy (Mendell et al., 2010). Additional AAV chimeras have also been developed to expand transduction to primary T cells, hematopoietic stem cells, and neurons (Bartel et al., 2011; Lisowski et al., 2014). Comparable strategies have been applied to lentiviral vectors, where the engineering of envelope glycoproteins, such as optimizing fusion junctions between RV-G and VSV-G (Kato et al., 2014) or dual pseudotyping with VSV-G and Sendai HN (Jargalsaikhan et al., 2024), has significantly enhanced neuronal gene transfer. Collectively, these studies highlight the versatility of structure-guided design across vector platforms and provide a rationale for applying similar strategies to PFV Env to improve its infectivity and cell-type targeting.

Foamy viruses (FVs) belong to the *Spumaretrovirinae* subfamily of the *Retroviridae* family and possess unique biological characteristics that distinguish them from other retroviruses (Linial, 1999; Khan et al., 2018; Lee et al., 2019; Simantirakis et al., 2020). FVs are endemic infectious agents in various mammalian species, including non-human primates, cats, cattle, and horses (Materniak-Kornas et al., 2020). Simian foamy viruses (SFVs) infect a wide range of non-human primates and can also be transmitted to humans without causing any apparent disease (Meiering and Linial, 2001; Lindemann and Rethwilm, 2011; Pinto-Santini et al., 2017). Prototype foamy virus (PFV) is a laboratory isolate originally recovered from a nasopharyngeal carcinoma culture and later shown to be derived from the eastern chimpanzee (*Pan troglodytes* schweinfurthii) SFV lineage. PFV shares characteristics with other retroviruses that integrate their viral genome into the host chromosome *via* an enzyme known as integrase (Lesbats et al., 2016; Kim et al., 2020). However, its integration profile is reported to be less likely to cause insertion mutations than gamma retrovirus or lentivirus vectors (Nowrouzi et al., 2006; Trobridge et al., 2006; Hocum et al., 2016). Furthermore, its non-pathogenicity, broad tropism, and large packaging capacity (∼12 kb) provide additional advantages in applicability (Erlwein and McClure, 2010; Sweeney et al., 2017; Meng et al., 2020). PFV has attracted increasing attention as an alternative gene delivery vector (Trobridge, 2009; Rajawat et al., 2019). However, despite these advantages, the relatively low transduction efficiency of PFV vectors in certain target cell types remains a barrier to broader clinical translation (Patton et al., 2004). In our previous study, we established a PFV dual-vector system and improved viral production and transgene expression by optimizing the codon usage of Env and incorporating the Woodchuck Hepatitis Virus Posttranscriptional Regulatory Element (WPRE) sequence under an empirically determined optimal packaging ratio (Cho et al., 2024).

Heparan sulfate (HS), a sulfated glycosaminoglycan abundantly displayed on mammalian cell surfaces, broadly contributes to viral engagement by functioning either as a direct attachment receptor that initiates infection or as a co-receptor that facilitates subsequent binding to the required entry receptors (Ravikumar et al., 2020; Hoffmann et al., 2022). Consistent with this general role, HS also serves as the primary attachment receptor for PFV, as demonstrated by studies showing that enzymatic removal or genetic depletion of HS markedly reduces viral entry (Nasimuzzaman and Persons, 2012; Plochmann et al., 2012). However, both studies also reported that PFV infection is not completely abolished under HS-deficient conditions, suggesting that HS is necessary for efficient attachment but may not fully account for all determinants of PFV infection. Although PFV Env engages HS during early entry, its precise structural determinants remain unclear (Duda et al., 2006; Nasimuzzaman and Persons, 2012; Plochmann et al., 2012). Recent crystal structure of simian FV Env has identified basic residues in the lower domain (LD) of the receptor-binding domain (RBD) as key mediators of HS binding (Fernandez et al., 2023). And cryo-EM analysis has resolved the overall architecture of PFV Env, demonstrating that it assembles into a trimeric class-I fusion complex in which the surface subunit (SU) presents a receptor-binding domain (RBD) at the trimer apex. The RBD is composed of an upper domain (UD) and a lower domain (LD), with the LD containing the basic surface implicated in HS engagement in simian foamy virus (SFV) (Fernandez et al., 2023; Calcraft et al., 2024). Together, these structural insights suggest that conserved structural features within the RBD LD are central to HS-dependent attachment in FVs.

Here, we present a structure-guided approach to dissect and re-engineer PFV Env to enhance HS-mediated viral entry. Using a combination of 3D modeling, molecular docking simulations, site-directed mutagenesis, and quantitative transduction assays, we analyzed the contributions of the upper and lower Env subdomains to viral attachment and infectivity. In parallel, we generated a chimeric Env by replacing the RBD of PFV with that of gorilla SFV (SFVgorII). Through targeted residue substitutions and domain swapping, we identified the structural features that govern HS interaction and improved transduction efficiency in selected engineered variants. Our data provide a rational framework for optimizing PFV vectors and contribute to the broader development of safer and more efficient gene therapy platforms.

## 2 Materials and methods

### 2.1 *In silico* molecular docking and mutant design

The amino acid sequence of Env, including its receptor-binding domain (RBD), is shown in Supplementary Figure 1. The PFV Env RBD was subsequently modeled using AlphaFold2 (AF2) software (Jumper et al., 2021). The modeled structure was validated by comparing the structure of RBD from SFV, indicating that PFV Env RBD has a comparable structural architecture to SFV Env RBD. The AlphaFold2 (AF2) modeled structure of the PFV Env RBD was validated using internal confidence metrics and structural comparison with the crystal structure of SFV RBD (PDB ID 8AEZ) (Fernandez et al., 2023). The predicted model showed a high overall confidence score with a predicted Template Modeling (pTM) score of 0.84. Structural alignment with the SFV RBD yielded a root-mean-square deviation (RMSD) of 0.725 Å over 220 pruned atom pairs (5.132 Å across all 328 pairs) (Supplementary Figure 2). To estimate the potential binding mode of heparan sulfate (HS), a docking simulation was conducted using Autodock-vina with the blind method (Eberhardt et al., 2021), as particular binding pockets had not yet been verified. To identify potential Heparan Sulfate (HS) binding sites on the PFV Env RBD without structural bias, we performed blind molecular docking using AutoDock Vina. The search space was defined to encompass the overall RBD structure including positively charged upper domain. A grid box set up with dimensions of 74.9 × 61.0 × 77.0 Å centered at −3.9, −5.6, −1.7. To ensure rigorous sampling of the conformational space and reliable identification of global energy minimum, the simulation was conducted with an enhanced exhaustiveness value of 12 with a random seed. The high-sampling protocol allowed for the confident prediction of energetically favorable binding poses within the defined search space. To refine the binding mode of HS within the lower domain (LD), the search grid was reduced to a focused volume of 37.3 × 49.6 × 31.0 Å centered at −1.8, −6.7, −9.5. The thirty docking poses were generated, and top ranked model was selected to guide mutagenesis of LD variants. To identify potential Heparan Sulfate (HS) binding sites on the chimeric Env RBD, we restricted the search space to LD. A grid box set up with dimensions of 28.1 × 27.5 × 29.7 Å centered at −28.3, −22.9, and 4.8 Å. The simulation was conducted with an enhanced exhaustiveness value of 12 with a random seed.

Further structural analysis for interaction interfaces between modeled PFV Env RBD and HS were conducted using Chimera-X (version 1.8) (Pettersen et al., 2021). Protein-ligand 2D interaction diagrams were generated using Maestro and SAMSON software package (OneAngstrom, 2025; Schrödinger, 2025). The structure of chimeric protein was modeled using Swiss-model and AF2 (Waterhouse et al., 2018; Jumper et al., 2021). After comparing the overall structure of the models, the AF2 model was chosen for the blind docking using Autodock-vina (Eberhardt et al., 2021).

### 2.2 Computational prediction of HS binding affinity and structural stability

To investigate the structural and functional properties of the heparan sulfate (HS) binding interface, we implemented an integrated *in silico* framework utilizing AlphaFold 3 (AF3) to generate high-resolution models of monomeric and trimeric envelope (Env) proteins for both the native Prototypic Foamy Virus (PFV) and the SFVgorII-PFV chimeric constructs (Abramson et al., 2024). The global folding stability (ΔG) of these structures was quantified using EvoEF2 (Huang et al., 2020), while the thermodynamic impacts of specific mutations (ΔΔG) were evaluated through DynaMut to ensure structural integrity (Rodrigues et al., 2021a). The stability of functional trimeric complexes was specifically assessed using mmCSM-PPI, providing a detailed analysis of multimer assembly competency (Rodrigues et al., 2021b). Binding affinities toward HS octasaccharides were characterized using a multi-algorithmic consensus approach, incorporating Vinascore, PRODIGY energy scores, and KDEEP deep-learning-based predictions (Jimenez et al., 2018). Predicted dissociation constants (pKd) were derived from KDEEP-calculated binding energies (ΔG) and expressed as Kd values (−log10 pKd) based on the thermodynamic relationship ΔG = *RT*ln(Kd) assuming a temperature T of 298.15 K. Finally, all computational metrics and experimental functional data were standardized *via* Z-score normalization and validated through Pearson correlation analysis.

### 2.3 Plasmid construction

To construct Env point mutation vectors (pCMV-Env upper domain (UD)/lower domain (LD) variants), a human codon-optimized Env-expressing plasmid was used as a backbone (Cho et al., 2024). When we constructed the pCMV-Env plasmid, we inserted a Kozak sequence before the *env* gene and added a Valine after the start codon. As a result, the amino acid numbers are shifted by one when compared to the reference sequence (Calcraft et al., 2024). The pCMV-Env vector was linearized with *Age*Ⅰ/*Xma*Ⅰ or *Age*Ⅰ/*Bam*HⅠ, followed by assembly with PCR-amplified Env point mutation fragments using the EZ-Fusion HT Cloning Kit (Enzyomics, Republic of Korea). To generate the PFV-SFV RBD chimeric Env constructs (wild type (WT) and variants), the codon-optimized SFV Env RBD sequence was synthesized and inserted into the pCMV-Env backbone using gene synthesis and cloning services provided by GenScript (USA). Env point mutation vectors were constructed using codon-optimized PFV Env as templates, with site-directed PCR performed using specific primers summarized in Supplementary Table 1; Mutation vectors not explicitly listed were generated either using combinations of the listed primers or *via* synthesis and cloning by GenScript (USA). All constructs were sequence-verified before their use.

### 2.4 Cell culture

The human fibrosarcoma cell line HT1080 was obtained from the Korean Cell Line Bank, and the human embryonic kidney cell line HEK293FT was obtained from Thermo Fisher Scientific (USA). Cells were maintained in Dulbecco’s modified Eagle’s medium (DMEM; Sigma-Aldrich, USA) supplemented with 10% fetal bovine serum (FBS; Gibco, USA), 1% penicillin/streptomycin (P/S; Sigma-Aldrich, USA), and 100 μg/mL Normocin (InvivoGen, USA) to prevent *mycoplasma* contamination. All cells were incubated at 37 °C in a humidified atmosphere containing 5% CO_2_.

### 2.5 PFV production and transduction

All transfections were performed using jetOPTIMUS (Polyplus, Germany) according to the manufacturer’s protocol. HEK293FT cells were seeded in six-well plates and transfected at 70%–80% confluency with 3 μg DNA, comprising the EGFP transgene vector (pcHFV-EF1α-EGFP_v3) and Env-expression vector at a ratio of 30:1 (Cho et al., 2024). Culture supernatants were collected 2–3 days post-transfection and filtered through a 0.45 μm syringe filter. For transduction, 1 mL of PFV-EGFP-containing supernatant was added to 1.0 × 10^6^ HT1080 cells per well in a six-well plate, with infection repeated three times at 2–2.5 h intervals.

For production using HEK293T-Tet-Env cell lines, cells were cultured in a six-well plate to 70%–80% confluency and transfected with 2.5 μg of the EGFP transgene vector. Doxycycline (Sigma-Aldrich, USA) was added at a final concentration of 2 μg/mL to induce Env expression. All subsequent steps, including supernatant collection and transduction of HT1080 cells, were performed as previously described (Cho et al., 2024).

### 2.6 Generation of Tet-On–inducible Env-expressing stable cell lines

The pLVX-TetOne-Puro-Env plasmid was constructed by replacing the EGFP sequence in the pLVX-TetOne-Puro-GFP (Addgene, cat#171123) backbone with the PFV Env coding region using a standard cloning procedure. For lentiviral particle production, HEK293FT cells were co-transfected with pLVX-TetOne-Puro-Env, pMD2.G (Addgene, cat#12259), and pCMV*Δ*R8.74 (Dull et al., 1998) at a 5:3:2 ratio (total 10 μg) using jetOPTIMUS. Supernatants were harvested 2–3 days later, filtered through a 0.45 μm syringe filter, and concentrated using a Lenti-X concentrator (Takara Bio USA, USA) following the manufacturer’s protocol. For stable line generation, HEK293T cells (1.0 × 10^6^ per well, six-well plate) were transduced with 0.6 mL of concentrated lentivirus supplemented with polybrene at a final concentration of 4 μg/mL (Santa Cruz Biotechnology, USA). Infection was performed twice at 12 h intervals. At 24 h after infection, 3 μg/mL Puromycin (InvivoGen, USA) selection was applied to establish a stable Tet-On–inducible Env-expressing cell population. The medium was replaced every 2 days to maintain the antibiotic concentration.

### 2.7 Fluorescence microscopy

EGFP fluorescence was visualized using an Axio Observer 3 inverted microscope equipped with a 475 nm excitation filter (Zeiss, USA). Images were acquired using an Axiocam 506 mono camera and processed using Zen 3.8 software (Zeiss, USA).

### 2.8 Flow cytometry

Three days post-transduction, cells were harvested and washed with 1X PBS by centrifugation at 1,600 rpm for 5 min at 4 °C, and then resuspended in 500 μL of ice-cold FACS buffer [1X PBS (pH 7.2) with 2% FBS, 2 mM EDTA, and 0.09% sodium azide]. Flow cytometry was performed using an Attune NxT Flow Cytometer (Thermo Fisher Scientific, USA), and the data were analyzed using FlowJo software (version 10.10.0; Tree Star, USA).

### 2.9 Western blotting

Cells were lysed in RIPA lysis buffer [150 mM NaCl, 1% non-ionic detergent, 1% sodium deoxycholate, 0.1% SDS] (Biomax, Republic of Korea), and the protein concentration was measured using a Qubit 4 fluorometer (Thermo Fisher Scientific, USA). Proteins (10 μg) were separated by SDS-PAGE and transferred to nitrocellulose membranes (Cytiva, USA). Membranes were blocked with Smart-Block^TM^ Fast Blocking Buffer (Biomax, Republic of Korea) and incubated overnight at 4 °C with rabbit polyclonal anti-Env antibody (1:500, AbClone, Republic of Korea) and rabbit monoclonal β-actin antibody (1:2,500, Cell Signaling Technology, USA). After washing, the membranes were incubated with goat anti-rabbit IgG-HRP secondary antibody (1:10,000, BioRad, USA) for 1–2 h at room temperature. Signals were detected using an E-blot chemiluminescence imaging system (e-BLOT Life Science, China). A rabbit polyclonal anti-Env antibody was custom-generated to specifically target the PFV Env-SU domains (AbClone, Republic of Korea).

### 2.10 Statistical analysis

Data are presented as mean ± Standard Error of Mean (SEM) from at least three independent experiments. Statistical significance was assessed using Student’s t-test (***p < 0.001, **p < 0.01, *p < 0.1, ns *p* > 0.1).

### 3.1 Structure-based mapping and functional mapping of HS-interacting residues in PFV Env

**FIGURE 1:**
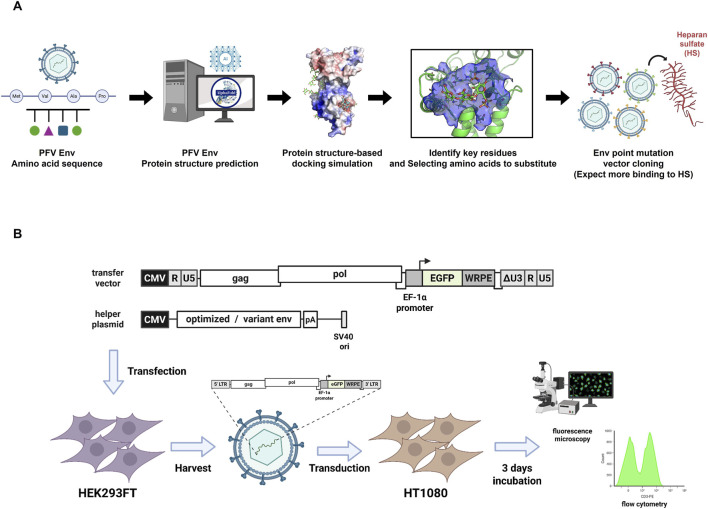
Schematic overview of structure-based engineering and functional validation of PFV Env mutants for enhanced heparan sulfate binding. **(A)** Structure-based strategy to identify key residues in the prototype foamy virus (PFV) Env protein responsible for heparan sulfate (HS) binding. The workflow begins with PFV Env amino acid sequence-based structure prediction, followed by docking simulations with HS to identify the interacting residues. Key amino acids predicted to mediate HS binding were selected and substituted to generate Env point mutants, which were subsequently cloned into expression vectors for functional testing. **(B)** Experimental workflow for PFV vector production and transduction assays. Transfer plasmid encoding EGFP reporter and Gag/Pol, together with the helper plasmid expressing either wild type or mutant Env were co-transfected into HEK293FT cells. The resulting viral particles were harvested and used to transduce the HT1080 cells. After 3 days of incubation, transduction efficiency and Env functionality were assessed using fluorescence microscopy, Western blotting, and flow cytometry.

To investigate the structural analysis of HS binding in PFV Env, we used an *in silico* prediction approach. A three-dimensional structural model of PFV Env was generated based on its amino acid sequence and subjected to molecular docking simulations using HS. Blind docking against the AF2-modeled PFV Env RBD identified three spatially separated regions accommodating potential HS binding. The docking predicts the most energetically favorable binding pose in a upper domain with binding free energy of −7.0 kcal/mol. In addition to the UD binding pockets, the lower domain showed cluster of predicted HS binding poses with binding energies range from −6.8 to −6.5 kcal/mol. Furthermore, a secondary, lower-affinity site was identified in the interface between the domains (rank 05, −6.0 kcal/mol) (Supplementary Figure 3). These distinct binding modes suggest alternative binding mode of HS towards PFV Env. Several candidate residues were predicted to directly interact with the negatively charged sulfate groups of HS. Based on these predictions, site-directed point mutations were designed at key residues and surrounding regions of the Env protein to enhance HS binding while preserving the protein’s overall structural integrity (Figure 1A).

To evaluate the transduction efficiency of the point-mutated Env, we followed a previously established PFV vector production and transduction workflow. HEK293FT cells were co-transfected with a transfer plasmid carrying the EGFP transgene and a helper plasmid expressing either WT or mutant Env. Viral supernatants were harvested and used to transduce HT1080 cells. After 3 days of incubation, the transduction efficiency was evaluated by monitoring EGFP expression using fluorescence microscopy. The transduction efficiency was quantitatively assessed across biological replicates using flow cytometry (Figure 1B). WT transduction efficiency varies between experimental sets because each panel was generated from an independent PFV production batch, and all analyses are normalized to the WT control produced in the same batch.

**FIGURE 2:**
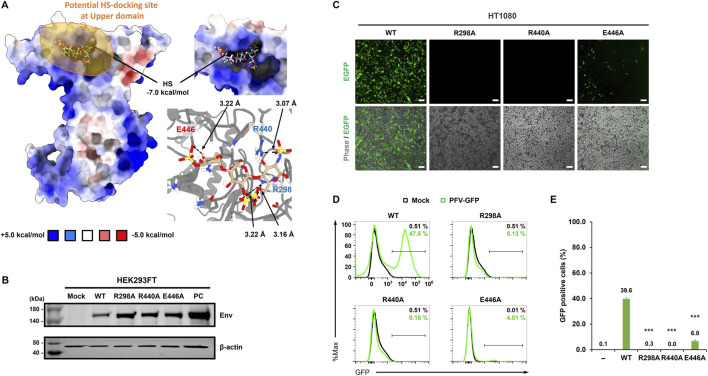
Functional characterization of PFV Env upper domain point mutants affecting HS binding and viral entry efficiency. **(A)** The structure shown represents the receptor-binding domain (RBD) of the PFV Env surface subunit (SU), modeled using AlphaFold2. The molecular docking pose of heparan sulfate (HS) is displayed on the positively charged pocket of the RBD upper domain (UD), with key interacting residues (R298, R440, and E446) highlighted. The binding free energy which calculated from Autodock-vina, −7.0 kcal/mol is labeled. The molecular docking simulation revealed three key residues, Arg(R)298, Arg(R)440, and Glu(E)446, located in UD. These residues form hydrogen bonding with negatively charged sulfate group of HS and are highlighted in red and cyan for negatively charged and positively charged residues, respectively. Based on this structural analysis, R298A, R440A, and E446A substitutions were designed to disrupt the HS binding. The distance between key residues and HS are labeled with black arrow. **(B)** Western blot analysis was performed to confirm the protein expression of wild type and Env variants (R298A, R440A, and E446A) in HEK293FT cells. The cells were co-transfected with the PFV transfer vector (v3) and Env plasmids at a 30:1 ratio. All three mutants showed Env protein levels comparable to those of the wild type, indicating that the point mutations did not affect protein expression or stability. *β*-actin was used as a loading control. Mock, HEK293FT cell line served as a negative control; PC, HEK293FT cells transfected pCMV-Env were used as a positive control. **(C)** To assess the effect of each Env variant on viral infectivity, supernatants from transfected HEK293FT cells were used to transduce HT1080 cells. Transduction efficiency was monitored by EGFP expression using fluorescence microscopy after 3 days. Phase-contrast images confirmed the equivalent cell density across conditions. Scale bars = 100 μm. **(D,E)** Flow cytometry was used to quantitatively measure the proportion of EGFP-positive HT1080 cells transduced with each viral construct. Representative histograms and bar graph quantifications (*N* ≥ 3) are shown in **(D)** and **(E)**, respectively. The average number from at least three independent experiments is shown at the top of each bar. Error bars represent the standard deviation of biological triplicates.

To identify potential HS-binding determinants, we performed an *in silico* prediction analysis of the entire Env RBD. This approach revealed a potential HS-docking site located within the upper domain (UD), with a calculated binding free energy of −7.0 kcal/mol, indicating a favorable interaction. Three residues (R298, R440, and E446) were predicted to be key candidates for forming electrostatic and hydrogen bond interactions with the negatively charged sulfate groups of HS (Figure 2A). To disrupt these interactions, we introduced alanine substitutions (R298A, R440A, and E446A). HEK293FT cells were co-transfected with the transfer plasmid pcHFV-EF1α-EGFP_v3 and either WT or mutant Env-expression plasmids. Western blot analysis confirmed that all three Env mutants were expressed at levels comparable to WT Env in transfected HEK293FT cells (Figure 2B), supporting that the introduced mutations did not affect protein stability or expression.

To analyze the transduction efficiency of the three mutants and compare them with WT (codon-optimized Env), we used the established PFV vector production and transduction workflow, as described in Figure 1B. Viral supernatants generated from HEK293FT cells were harvested and used to transduce HT1080 cells. At 3 days post-transduction, EGFP expression was monitored using fluorescence microscopy (Figure 2C), and the proportion of EGFP-positive cells was quantitatively measured using flow cytometry (Figures 2D,E). While WT Env mediated efficient transduction (39.6%), the R298A (0.3%) and R440A (0.0%) substitutions caused a complete loss of infectivity. The E446A mutant (6.8%) also exhibited a markedly reduced transduction efficiency compared to that of WT, indicating a severe loss of function. Collectively, these results demonstrate that R298, R440, and E446 are critical determinants of HS binding, which underlies PFV entry and gene delivery.

### 3.2 Structure-guided mutagenesis around HS-contacting residues of the upper domain enhances transduction efficiency

**FIGURE 3:**
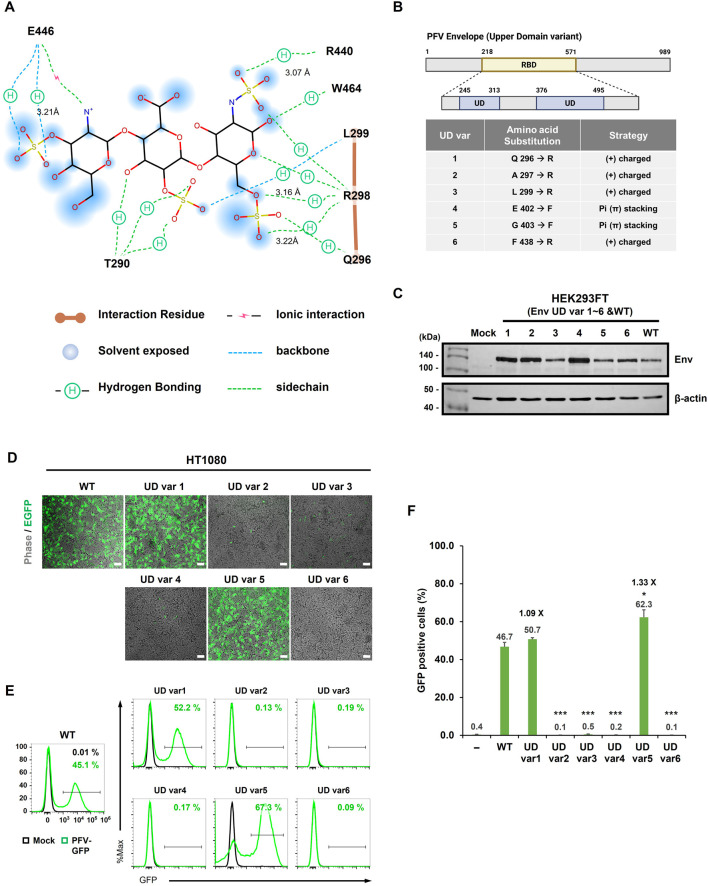
Structure-guided refinement of PFV Env mutants to dissect the HS-binding determinants in the upper domain. **(A)** Two-dimensional interaction diagram showing the predicted hydrophobic and electrostatic interactions between selected residues and HS sulfate groups. **(B)** Schematic representation of the domain organization of PFV Env (1–988 aa), highlighting the receptor-binding domain (RBD; aa 217–570), including upper domains (UD; aa 244-312, 375-494) based on structural predictions. **(C)** Western blot validation of Env protein expression in HEK293FT cells transfected with the PFV transfer vector and one of the six-point mutant Env constructs. The co-transfection ratio (30:1) was consistent with that of previous experiments. *β*-actin served as a loading control. Mock, HEK293FT cell line served as a negative control. **(D)** Fluorescence microscopy showing the differential infectivity of PFV vectors with each Env variant in HT1080 cells, as measured by EGFP expression. Phase-contrast images confirmed similar cell confluency across all conditions. Scale bars = 100 μm. **(E,F)** Flow cytometry was used to quantitatively measure the proportion of EGFP-positive HT1080 cells transduced with each viral construct. Representative histograms and bar graph quantifications (*N* ≥ 3) are shown in **(E,F)**, respectively. The average number from at least three independent experiments is shown at the top of each bar. Data are presented as mean ± SEM of biological triplicates.

Next, we engineered six UD variants by targeting residues adjacent to the predicted HS-binding sites using two design strategies: introduction of positively charged residues to enhance electrostatic attraction (Q296R, A297R, L299R, and F438R) and substitution with aromatic residues to promote *π-π* stacking with HS (E402F and G403F) (Figures 3A,B). All six variants were robustly expressed in HEK293FT cells at levels comparable to those of WT Env (Figure 3C), indicating these substitutions did not compromise protein expression or stability.

Upon transduction of HT1080 cells, only UD var1 (Q296R) and var5 (G403F) supported detectable infection, as evidenced by EGFP fluorescence microscopy, whereas UD var2 (A297R), var3 (L299R), var4 (E402F), and var6 (F438R) showed little or no detectable signal (Figure 3D). Flow cytometry confirmed these results, showing that viral vectors carrying WT Env yielded 44.0% EGFP-positive cells, whereas UD var1 (Q296R) and var5 (G403F) reached 52.2% and 67.3%, respectively. In contrast, UD var2/3/4/6 were essentially nonfunctional (<0.5%) (Figures 3E,F). Collectively, these data demonstrate that specific substitutions within the UD-HS interface enhance PFV transduction efficiency. The introduction of a positive charge at Q296 or an aromatic residue at G403 moderately improved viral entry, whereas substitutions at neighboring residues were not tolerated.

### 3.3 Structure-guided mutagenesis of the lower domain and cross-domain mutational synergy improves PFV transduction efficiency

Recent structural work on SFV Env suggested that several HS interacting residues reside within the LD of the receptor-binding domain (Fernandez et al., 2023). To test whether analogous positions in PFV Env contribute to HS-dependent entry, we introduced charge-enhancing substitutions at the corresponding PFV LD sites and evaluated their effects on transduction efficiency. However, these substitutions did not enhance entry and instead reduced transduction relative to WT, indicating that the SFV-derived HS-binding motif does not directly translate into functional enhancement within the PFV background (Supplementary Figure 4).

**FIGURE 4:**
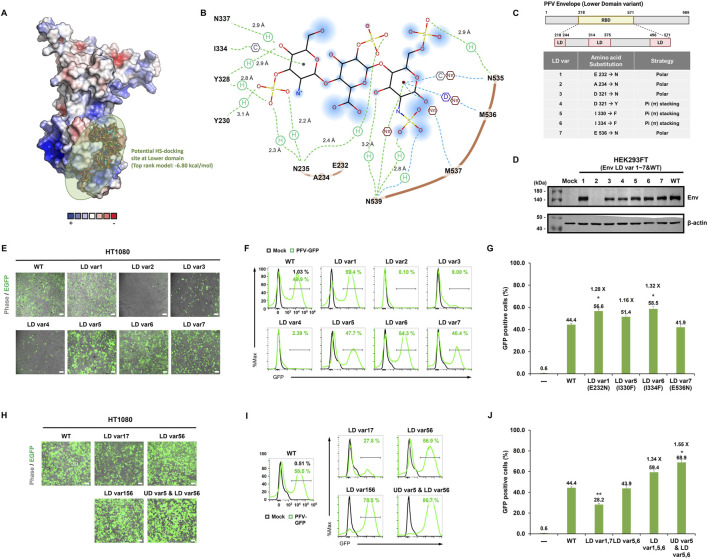
Functional screening of lower-domain Env variants. **(A)** Molecular docking simulation of the PFV Env RBD, highlighting candidate HS-binding residues in the lower domain (circled green). **(B)** Two-dimensional interaction diagram illustrating predicted hydrophobic and electrostatic interactions between selected lower domain residues and HS sulfate groups. **(C)** Schematic representation of PFV Env organization (1-988 aa), showing the localization of lower domain (LD) subregions (aa 217-243, 313-374, and 495-570), as determined by structural modeling. Guided by structure-based docking, seven candidate residues within the LD were selected for mutagenesis based on side-chain polarity and the potential for π-stacking interactions to enhance HS binding. The specific amino acid substitutions and their rationales are listed. **(D)** Western blot analysis of Env expression in HEK293FT cells co-transfected with vectors encoding each LD point mutant and the PFV packaging system. Mock, HEK293FT cell line served as a negative control. **(E,F)** EGFP fluorescence **(E)** and corresponding flow cytometry histograms **(F)** of HT1080 cells transduced with PFV particles carrying individual LD variants and representative flow cytometry histograms corresponding to these samples. **(H,I)** EGFP fluorescence imaging **(H)** and flow cytometry histograms **(I)** of HT1080 cells transduced with PFV particles containing double (LD var1,7; LD var5,6) or triple (LD var1,5,6; UD var5 and LD var5,6) variants. **(G,J)** Quantification (*N* ≥ 3) of GFP-positive HT1080 cells by flow cytometry, summarizing means ± SEM from biological triplicates.

Although substituting PFV LD residues with the known SFV HS-binding residues did not improve transduction efficiency, we sought to determine whether the PFV LD nonetheless harbors a potential HS-binding site. Thus, we performed molecular docking simulations, which revealed a candidate binding pocket on the LD of Env that is compatible with HS interactions (Figure 4A). Seven residues in this pocket (LD var1-7: E232N, A234N, D321N, D321Y, I330F, I334F, and E536N) were selected for mutagenesis using two design strategies: (1) substitution with polar, uncharged residues, such as asparagine, to facilitate hydrogen bonding while avoiding the steric hindrance predicted for bulky positively charged residues (Arg/Lys), and (2) substitution with aromatic residues to enable π-π stacking with HS (Figures 4B,C). Unlike the UD mutagenesis strategy, where positive charges are introduced to reinforce electrostatic attraction (Figures 3A,B), the LD design focuses on polarity- and stacking-based substitutions to preserve structural compatibility with the local binding environment. All seven variants (LD var1-7) were expressed at levels comparable to WT Env in HEK293FT cells, indicating no disruption in protein expression or stability (Figure 4D).

Fluorescence imaging and flow cytometry of HT1080 cells transduced with PFV variants revealed differential effects on transduction efficiency (Figures 4E–G). Of the single mutants, LD var1 (E232N), LD var5 (I330F), and LD var6 (I334F) exhibited enhanced transduction efficiency compared to WT, yielding 56.6%, 51.4%, 58.5% EGFP-positive cells, respectively. Compared to WT (44.4%), these mutants achieved up to 1.25-fold enhancement in transduction efficiency. In contrast, other variants, LD var2 (A234N), var3 (D321N), var4 (D321Y), and var7 (E536N), displayed markedly reduced transduction (<10%).

To explore the potential synergistic effects, we generated combinatorial variants with multiple beneficial variants. The triple mutant LD var1+5+6 and UD var5 with LD var5+6 further improved EGFP expression, reaching 59.4% and 68.9% transduction efficiencies, respectively (Figures 4H–J). These values corresponded to ∼1.25-fold and ∼1.55-fold increases compared to those of WT (44.4%). In one of the repeated experiments, UD var5 with LD var5+6 achieved transduction efficiencies as high as 86.7% (Figure 4I), underscoring the strong synergistic effect of these mutations.

These results demonstrate that rational substitution of specific LD residues can enhance PFV-mediated gene delivery, and suggest that local side-chain properties, including the potential to favor π–π stacking interactions, may contribute to these functional effects. Furthermore, combining beneficial mutations across both the UD and LDs maximizes transduction efficiency, offering a promising strategy for Env engineering.

### 3.4 Impact of interspecies domain swapping on structural compatibility

**FIGURE 5:**
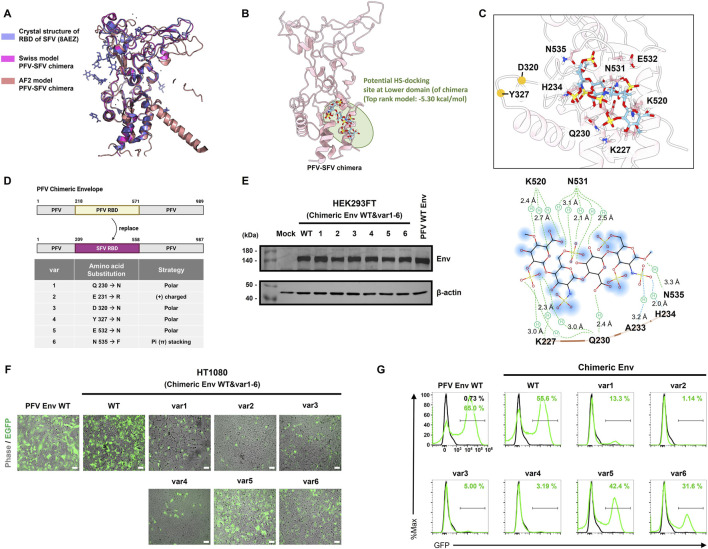
Structure-based functional screening of chimeric PFV-SFV Env variants to identify critical residues in HS-mediated viral entry. **(A)** Structural alignment of the SFV RBD crystal structure (PDB: 8AEZ) with the PFV-SFV gorⅠⅠ chimera RBD predicted by Swiss-Model and AlphaFold2 (AF2). **(B)** Molecular docking simulation of the PFV-SFV chimera RBD, highlighting the potential HS-binding residues in the lower domain (circled in green). **(C)** Zoomed view of the interaction interfaces and key residues (upper panel) and two-dimensional interaction diagram (bottom panel) showing predicted hydrophobic and electrostatic interactions between selected residues and HS sulfate groups. **(D)** Schematic representation of the PFV Env domain organization, with the SFV gorⅠⅠ RBD (aa 208-557) replacing the PFV RBD (aa 217-570). Specific amino acid substitutions (var1-6) and their rationales are listed. **(E)** Western blot analysis confirmed the expression of wild type chimeric Env (PFV backbone with SFV RBD) and six chimeric Env point mutants (variants 1–6) in the HEK293FT cells. β-actin was used as a loading control. PFV wild type Env (PFV WT Env)-transfected HEK293FT cells were used as a positive control. Mock, HEK293FT cell line served as a negative control. **(F)** Infectivity of each construct was assessed by EGFP fluorescence in HT1080 cells transduced with the PFV vectors. **(G)** Flow cytometry analysis quantified the proportion of GFP-positive cells for PFV WT Env, chimeric WT Env, and chimeric Env.

To investigate whether interspecies structural variations in the RBD influence HS-mediated entry, we engineered a chimeric PFV Env in which the native PFV RBD (aa 218-571) was replaced with that of gorilla SFVgorⅠⅠ (aa 209-558). Structural alignment of the PFV-SFV chimeric RBD, modeled using Swiss-Model and AlphaFold2, revealed substantial overall conservation with the SFV RBD (PDB: 8AEZ) (Figure 5A). Despite this global similarity, distinct conformational differences have been observed in the surface-exposed, flexible loop regions, which are often implicated in receptor interactions (Yuan et al., 2013; Buning and Srivastava, 2019). These observations prompted us to construct and assess a chimeric Env.

Molecular docking simulations identified a potential HS-binding site located in the LD of chimeric Env (Figure 5B). Based on interaction mapping between HS and SFVgorⅠⅠ RBD, we introduced six targeted amino acid substitutions into the chimeric Env construct (Figures 5C,D). These include polar uncharged substitutions (Q230N, D320N, Y327N, and E532N), positively charged substitutions (E231R), and aromatic substitutions (N535F). Western blot analysis confirmed the robust expression of PFV WT Env, chimeric WT Env, and all six variants in HEK293FT cells, with protein levels comparable to those of PFV WT Env (Figure 5E).

EGFP fluorescence imaging and flow cytometry analysis revealed substantial differences in transduction efficiency across the constructs (Figures 5F,G). PFV WT Env mediated 65.0% EGFP-positive cells, whereas the chimeric WT Env construct reached 55.6%, indicating that the chimeric Env is functional but less efficient than PFV WT. Among the variants, var5 (E532N) and var6 (N535F) retained moderate activity (42.4% and 31.6%, respectively), whereas the remaining substitutions (var1-4) resulted in markedly reduced infectivity (<13.3%). Both chimeric Env WT and its variants displayed reduced transduction efficiency compared to PFV WT Env, suggesting that domain replacement is partially compatible but may compromise optimal receptor engagement or membrane fusion dynamics.

### 3.5 Establishment of Tet-On–inducible PFV Env-expressing HEK293T cell lines

**FIGURE 6:**
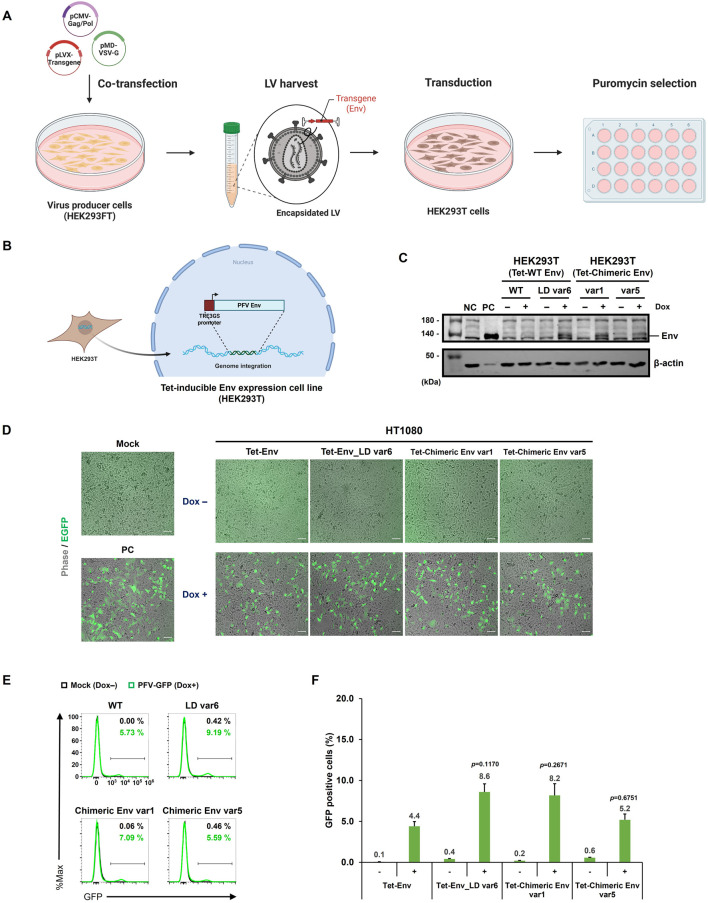
Establishment and functional validation of Tet-On–inducible PFV Env-expressing HEK293T cell lines. **(A)** Workflow for generating stable cell lines. HEK203FT virus producer cells were co-transfected with three plasmids (pCMV-Gag/Pol, pMD-VSV-G, and pLVX-Transgene). Lentiviral particles carrying the transgene were harvested and used to transduce the HEK293T cells. Stable cell populations were established through puromycin selection. **(B)** Schematic representation of the Tet-On doxycycline (Dox)-inducible system used to drive PFV Env expression in HEK293T cells. The Env cassette was stably integrated into the lentiviral vectors. **(C)** Western blot analysis confirmed the establishment of HEK293T cells harboring Tet-Env cassettes. Cell lysates were analyzed by Western blotting using anti-Env and anti-β-actin antibodies. The letters above the lanes indicate Env type and Dox treatment. NC, HEK293FT cell line served as a negative control; PC, PFV wild type Env (PFV WT Env)-transfected HEK293FT cells were used as a positive control. **(D)** Functional validation using an infection assay. HT1080 cells were infected with viral particles produced from Tet-Env-293T clones following Dox induction. Successful production of infectious viruses was confirmed by GFP expression observed under fluorescence microscopy. **(E)** Flow cytometry analysis of HT1080 cells infected with supernatants from individual TetEnv-293T clones with or without Dox treatment. **(F)** Quantification (*N* ≥ 3) of GFP-positive HT1080 cells by flow cytometry, summarizing means ± SEM from biological triplicates.

To enhance reproducibility and enable the scalable production of PFV-based vectors, we established a panel of tetracycline (Tet)- inducible PFV Env-expressing HEK293T cell lines. Lentiviral vectors encoding PFV Env were transduced into HEK293T cells, and stable populations were selected following puromycin treatment (Figure 6A). The Env transgene cassettes were integrated under the control of the TRE3GS promoter, allowing for tight transcriptional regulation by doxycycline (Dox) (Figure 6B).

In addition to the WT Env construct, we generated cell lines incorporating a lower-domain mutant (LD var6) and two chimeric Env variants (var1 and var5). To verify the expression of these constructs, we performed Western blot analysis on the established clones. Western blotting confirmed negligible Env expression in the absence of Dox and robust induction following Dox treatment across all variants (Figure 6C), with a minimal background signal, confirming the fidelity of the inducible system.

To determine whether Dox-induced Env expression was sufficient to generate infectious PFV particles, supernatants from 293T-Tet-Env clones were collected and used to infect HT1080 cells. Fluorescence microscopy revealed GFP expression exclusively in the Dox-induced groups, indicating productive viral particle formation upon Env induction (Figure 6D). Flow cytometry was used to quantify the transduction efficiency (Figures 6E,F). While WT Env-expressing cells achieved an average of 4.4% GFP-positive HT1080 cells, Tet-Env LD var6 showed the highest transduction efficiency (8.6%), followed by Tet-chimeric Env var1 (8.2%).

Because overall transduction efficiency from stable cell lines was lower than that observed with transient Env expression, we tested whether transcript stabilization could improve Env output. For this, we introduced the human β-globin intron (hBGi) upstream of the Env coding region, as intron inclusion has been shown to enhance mRNA stability and transgene expression in viral vector systems (Antoniou et al., 1998; Kang et al., 2005; Haddad-Mashadrizeh et al., 2009). Western blot analysis demonstrated stronger inducible Env expression in Tet-Env_hBGi cells compared to WT (Supplementary Figure 5A). Viral supernatants from Tet-Env_hBGi mediated enhanced GFP expression in HT1080 cells (Supplementary Figure 5B), with flow cytometry showing 8.1% GFP-positive cells compared to 4.4% for WT Env (Supplementary Figures 5C,D).

Together, these results demonstrate the successful establishment of a Tet-On inducible PFV Env expression system in HEK293T cells and its utility for functional evaluation of engineered Env variants. Inducible expressions of WT, LD var6, and chimeric Env variants resulted in detectable production of infectious PFV particles, with LD var6 showing the highest efficiency. Because the overall infectivity obtained from the inducible Env-expressing cells remains lower than that achieved under transient overexpression, strategies that increase Env expression are required to improve the performance of the stable producer system. Incorporation of the human β-globin intron (hBGi) further enhanced transcript stability and Env expression, leading to improved transduction compared to the WT control. Thus, Figure 6 and Supplementary Figure 2 collectively validate the inducible platform as a reproducible system for Env production while also highlighting strategies, such as hBGi inclusion, that can partially overcome the reduced efficiency typically observed in stable producer cell lines.

## 4 Discussion

In this study, we aimed to define the structural determinants of HS binding within PFV Env and evaluate whether rational engineering could enhance viral entry and gene transfer efficiency. Using a combination of *in silico* prediction, molecular docking, and systematic mutagenesis across the upper and lower domains of the Env RBD, we generated a comprehensive panel of Env variants and assessed their infectivity in cell-based assays. The outcomes of this mutational analysis are summarized in Supplementary Figure 6 and Supplementary Table 2. Although the magnitude of improvement was modest compared to the dramatic gains reported in AAV or lentiviral systems, several PFV Env variants nonetheless achieved reproducible 1.09–1.55-fold increases in transduction efficiency. These increases are biologically meaningful given that PFV generally exhibits modest baseline transduction efficiency in adherent cell lines such as HT1080. The observed enhancements arise from highly specific reinforcement of local electrostatic or π–π interactions that strengthen HS engagement while preserving the structural integrity of the RBD. Moreover, because PFV entry displays marked cell-type dependence, variants that enhance transduction in HT1080 cells may yield equal or even greater improvements in other cellular contexts with differing HS density or cofactor expression profiles. Thus, the fold increases reported here likely represent a conservative estimate of the maximal functional potential of these variants. Accordingly, systematic evaluation of Env variant–dependent transduction efficiency across diverse cell types is an essential next step, with particular emphasis on therapeutically relevant target cells for gene delivery applications. Finally, as no prior structure-guided mutational analysis of PFV Env has been reported, these findings constitute the first experimentally validated gain-of-function Env mutations shown to enhance PFV entry. In this context, even modest improvements provide foundational mechanistic insight into how specific side-chain chemistries modulate PFV–HS interactions and offer a rational starting point for further engineering of PFV-based gene delivery systems.

Nonetheless, our analysis provides important insights into the structural logic of PFV Env-HS interactions. Alanine substitutions at the canonical HS-contacting residues abolished infectivity, whereas strategic substitutions at neighboring positions, particularly Q296R in the UD and E232N, I330F, and I334F in the LD, enhanced transduction, suggesting that local electrostatic and *π-π* stacking interactions can be exploited to strengthen Env-HS binding. Docking simulations further predicted the strongest HS-binding energies on the UD, and the observation that certain UD substitutions completely eliminated entry supports the view that the UD provides essential structural or electrostatic features required for HS recognition. In contrast, substitutions within the LD produced several gain-of-function variants, indicating that the LD contributes a tunable HS-contacting surface capable of modulating attachment efficiency. Together, these results show that neither domain alone is sufficient to define HS binding; rather, the UD and LD act cooperatively to form the composite HS-recognition interface. Importantly, attempts to mimic SFVgorII key residues in the PFV background reduced infectivity, underscoring that HS-binding determinants are not universally transferable across foamy virus Envs and highlighting the necessity of residue-level, context-dependent engineering. This domain cooperativity ultimately motivated our combinational design strategy, in which beneficial UD and LD mutations were integrated to achieve maximal enhancement of PFV transduction.

We have conducted comprehensive theoretical analysis to accurate prediction of binding affinity toward HS on the AlphaFold three modeled PFV RBD and its variants (Supplementary Figures 7A–C) by using multiple algorithms including vina score, PRODIGY energy score, KDEEP score and calculated Kd from KDEEP energy (Supplementary Figure 7D; Supplementary Table 3). Although experimental and functional outcomes are not fully linked with the binding affinity between HS (Supplementary Figure 7D), overall Pearson correlation analysis showed that KDEEP score (ΔG) and predicted Kd (pKd) from KDEEP prediction have moderate correlation with functional outcome (GFP positive) as *r* = 0.73 and *r* = −0.73, respectively (Supplementary Figure 7E). Since the low binding affinity has high z-score, there is no negative correlation between binding affinity and functional outcome (Supplementary Figure 7E; Supplementary Table 3). The calculation of binding affinity of HS toward chimeric Env protein also showed no correlation between binding affinity and functional outcome (Supplementary Figure 8). To investigate the structural integrity and structural impact of each variant, we then performed prediction of fold stability and the mutational effects on thermodynamic stability as well as multimer stability. Interestingly, most of PFV variants showed improved stability with low Z-score except Q296R (UD var01), D321Y (LD var04), and E536N (LD var07) (Supplementary Figures 9A,B). However, it does not show any of correlation between ΔΔG calculation and its transduction efficiency. The variants relevant for chimeric Env showed that lowered stability (ΔΔG) with high Z-score (Supplementary Figures 9C,D). The decreased stability might hinder the decreased transduction efficiency despite of high binding affinity of chimeric variants (Supplementary Figures 8, 9). The fold stability of WT proteins in current study, PFV Env, chimeric Env, and SFV Env showed moderate difference, and it supported the stable expression of the WT proteins (Supplementary Figures 9A,C,E). In addition, most of the UD variants of PFV Env showed moderate effects on the stable formation of functional trimeric complex (Supplementary Figure 9F) and effects of LD variants were not determined (Supplementary Figures 9F,G). We established an integrated *in silico* framework, combining AlphaFold 3 modeling with multi-algorithmic affinity scoring, to characterize both heparan sulfate (HS) binding thermodynamics and the structural stability of the resulting functional complexes. Our analysis revealed a paradoxical positive correlation between predicted binding energy (ΔG) and transduction efficiency (*r* = 0.73), suggesting that the relationship between attachment affinity and viral entry is non-linear. Consequently, high-resolution molecular dynamics (MD) simulations and direct biophysical kinetic analyses are essential to resolve the specific transitions governing productive protein design.

In addition, the establishment of Tet-On–inducible PFV Env-expressing HEK293T cell lines (Figure 6; Supplementary Figure 5) provides a standardized platform for evaluating engineered Env variants under controlled conditions. Although the transduction efficiencies achieved with these stable cell lines were lower than those obtained with transiently produced vectors, likely due to reduced Env expression levels in producer cells, the system offers clear advantages in reproducibility, scalability, and consistency. Notably, incorporation of the human β-globin intron (hBGi) improved Env transcript stability and enhanced transduction efficiency nearly twofold compared to WT Env in the stable cell system. These findings suggest that combining transcript-stabilizing elements such as hBGi with beneficial Env variants (e.g., LD var6 or combinatorial variants) may help overcome the limitations of stable producer cells and enable more efficient PFV-mediated gene transfer in future applications. PFV vectors are further distinguished by their broad tropism and non-pathogenicity, yet their transduction efficiency remains cell type-dependent, which may explain the modest gains observed in our engineered variants. Future studies will therefore extend these analyses to additional cell types, as the relative performance of individual Env variants is likely to vary with cellular context and receptor landscape.

## Data Availability

The original contributions presented in the study are included in the article/Supplementary Material, further inquiries can be directed to the corresponding authors.
