# Supplementary material for: Structure-guided engineering of prototype foamy virus Env identifies key residues for heparan sulfate binding and enhances transduction efficiency
Source: Front Bioeng Biotechnol. 2026 Jan 26;14:1716928. doi: 10.3389/fbioe.2026.1716928 (PMC12883778; doi:10.3389/fbioe.2026.1716928)
Supplement: Supplementary file 1 [file Supplementaryfile1.docx]

Supplementary Material

# Supplementary Figures


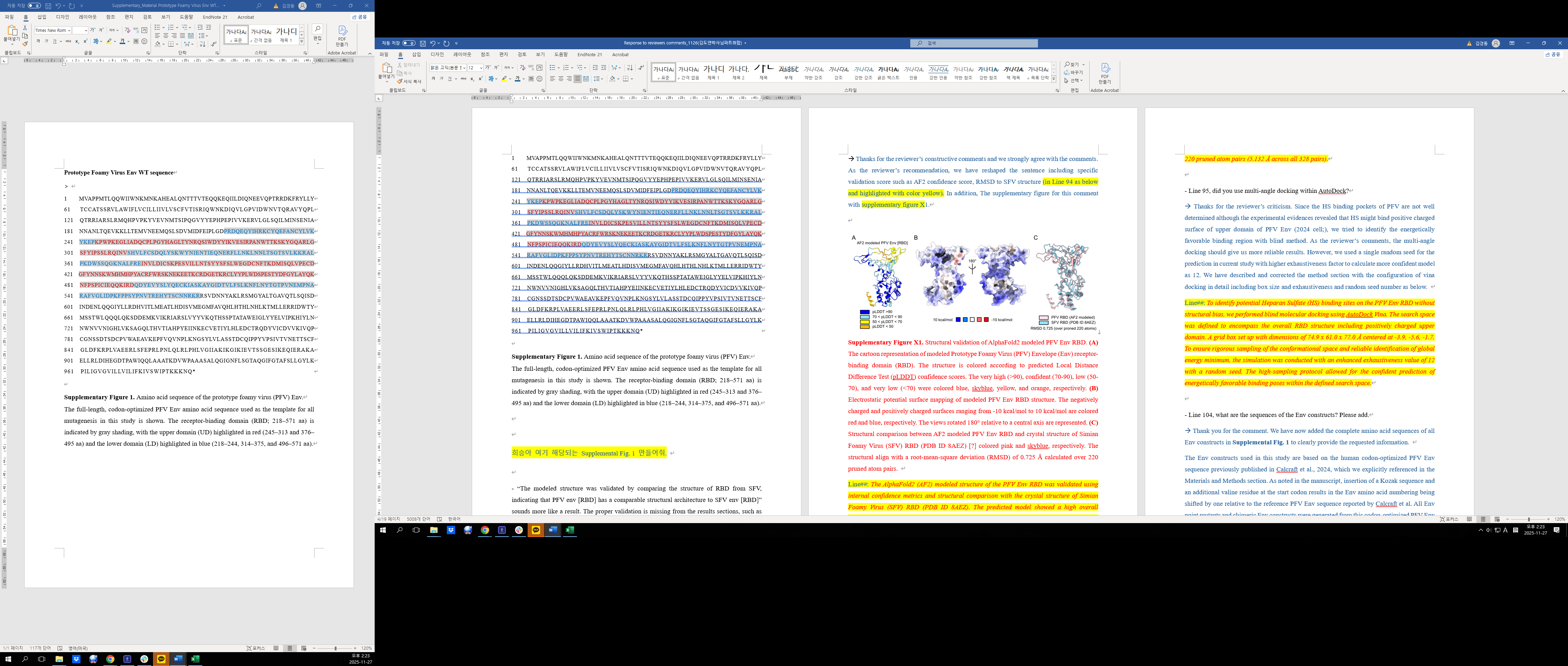


**Supplementary Figure 1.** Amino acid sequence of the prototype foamy virus (PFV) Env. The full-length, codon-optimized PFV Env amino acid sequence used as the template for all mutagenesis in this study is shown. The receptor-binding domain (RBD; 218–571 aa) is indicated by gray shading, with the upper domain (UD) highlighted in red (245–313 and 376–495 aa) and the lower domain (LD) highlighted in blue (218–244, 314–375, and 496–571 aa).


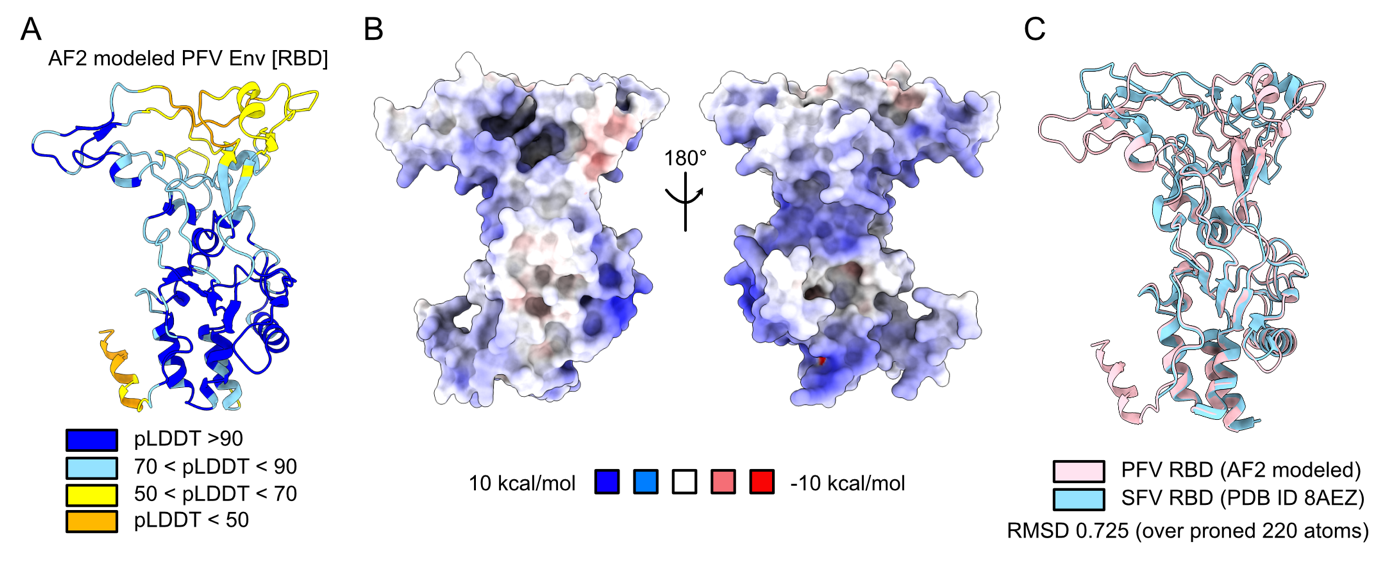


**Supplementary Figure 2.** Structural validation of AlphaFold2 modeled PFV Env RBD. **A**. The cartoon representation of modeled Prototype Foamy Virus (PFV) Envelope (Env) receptor-binding domain (RBD). The structure is colored according to predicted Local Distance Difference Test (pLDDT) confidence scores. The very high (>90), confident (70-90), low (50-70), and very low (<70) were colored blue, skyblue, yellow, and orange, respectively. **B**. Electrostatic potential surface mapping of modeled PFV Env RBD structure. The negatively charged and positively charged surfaces ranging from -10 kcal/mol to 10 kcal/mol are colored red and blue, respectively. The views rotated 180° relative to a central axis are represented. **C**. Structural comparison between AF2 modeled PFV Env RBD and crystal structure of Simian Foamy Virus (SFV) RBD (PDB ID 8AEZ) (Fernandez et al., 2023) colored pink and skyblue, respectively. The structural align with a root-mean-square deviation (RMSD) of 0.725 Å calculated over 220 pruned atom pairs.


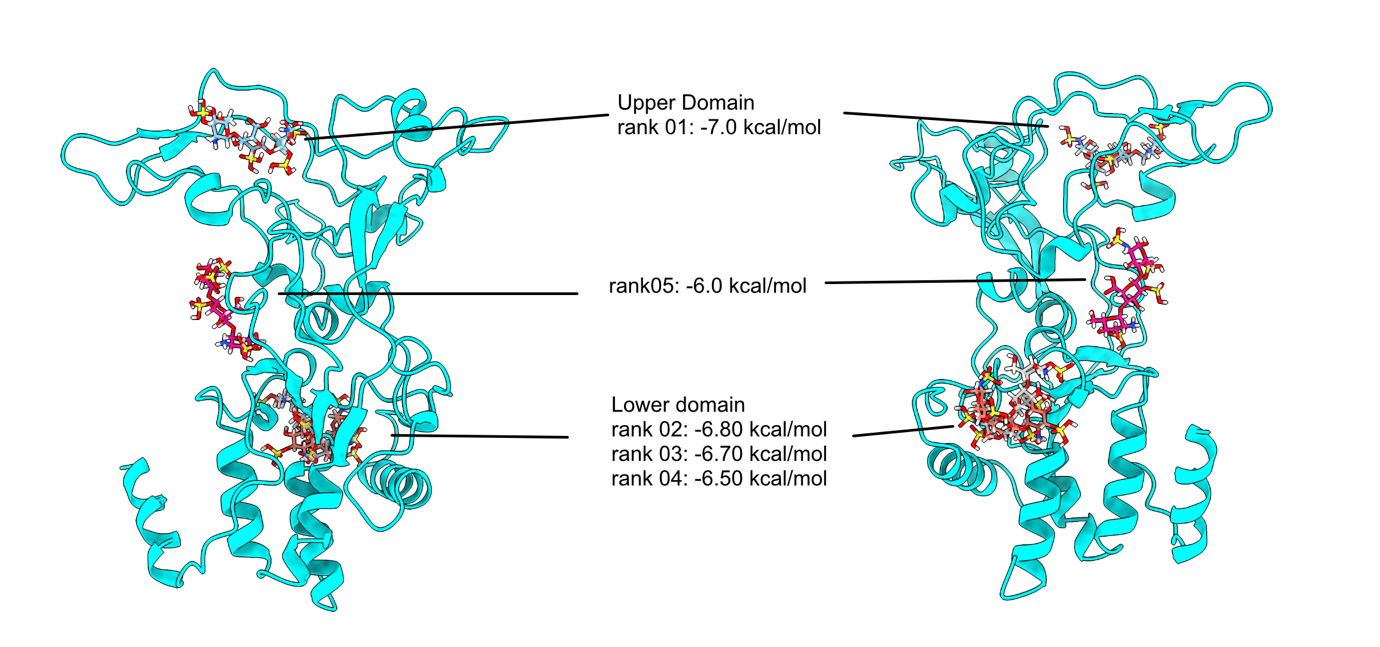

**Supplementary Figure 3.** Blind docking simulation and energy ranking of HS binding poses against PFV Env RBD. The AF2-modeled PFV Env RBD is represented as cartoon diagram shown in two different orientations to visualize potential HS binding sites. The top-ranked ligand poses are represented by stick models. The calculated binding free energy (ΔG in kcal/mol) for binding poses are labeled.


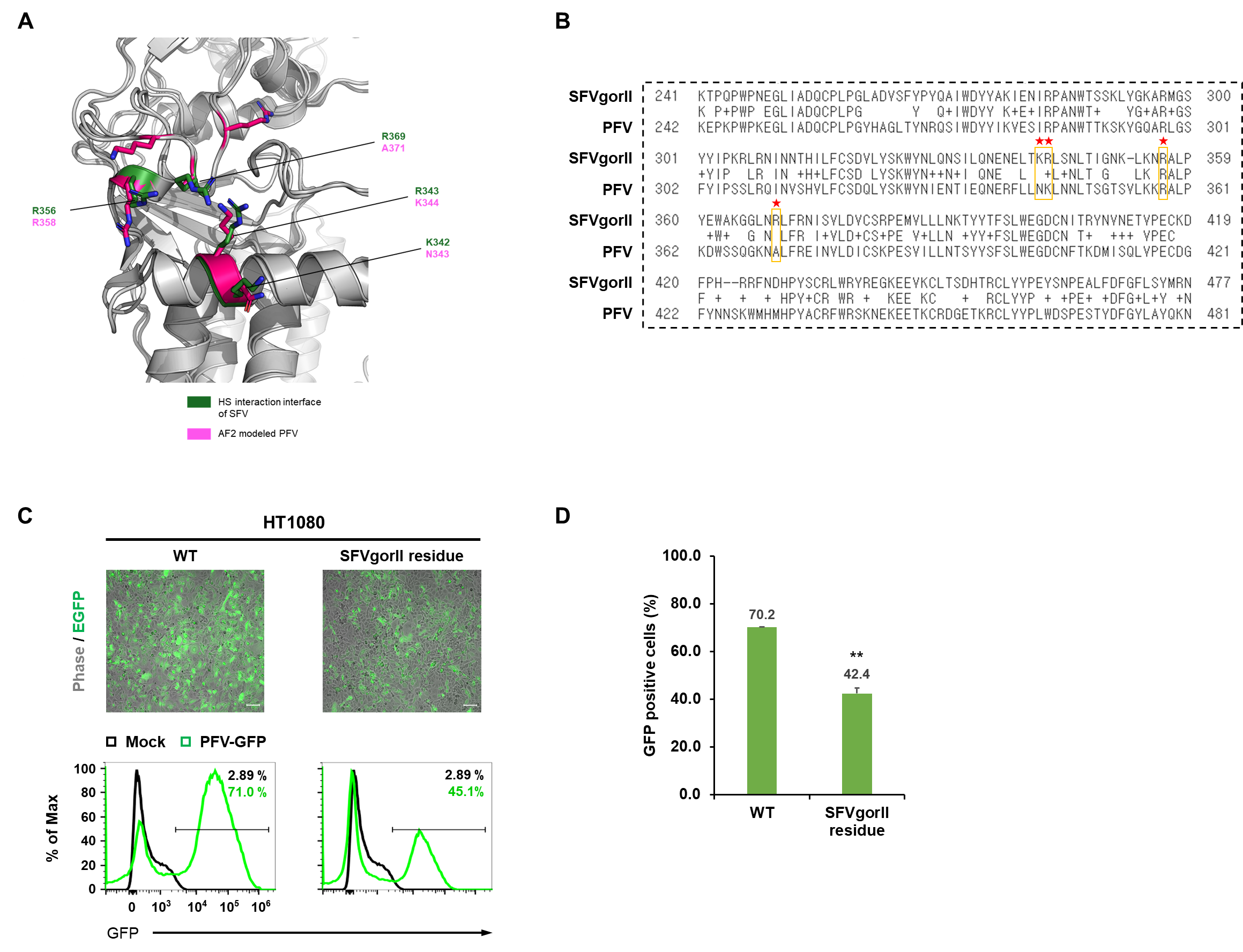


**Supplementary Figure 4.** Mapping SFVgorⅠⅠ HS-interaction residues onto PFV Env and functional validation by residue substitution. We hypothesized that the LD of PFV Env may also contribute to HS interactions and serve as a rational target for engineering to enhance cell entry. **A**. Superimposition of the LD structures of SFV Env and PFV Env revealed a high degree of structural similarity between the two proteins. **A, B**. The residues identified as key for HS binding in SFV Env (K342, R343, R356, and R369) aligned with the corresponding positions in PFV Env (N342, K343, R357, and A370). **C, D**. To test whether mimicking the SFV configuration would enhance HS interactions, we substituted PFV residues with lysine and arginine at the equivalent sites. However, functional assays revealed these substitutions, rather than enhancing infectivity, markedly reduced transduction efficiency compared to WT Env, indicating that the LD of PFV Env is not functionally interchangeable with that of SFV Env and that the local residue context is critical for maintaining entry competence.

**
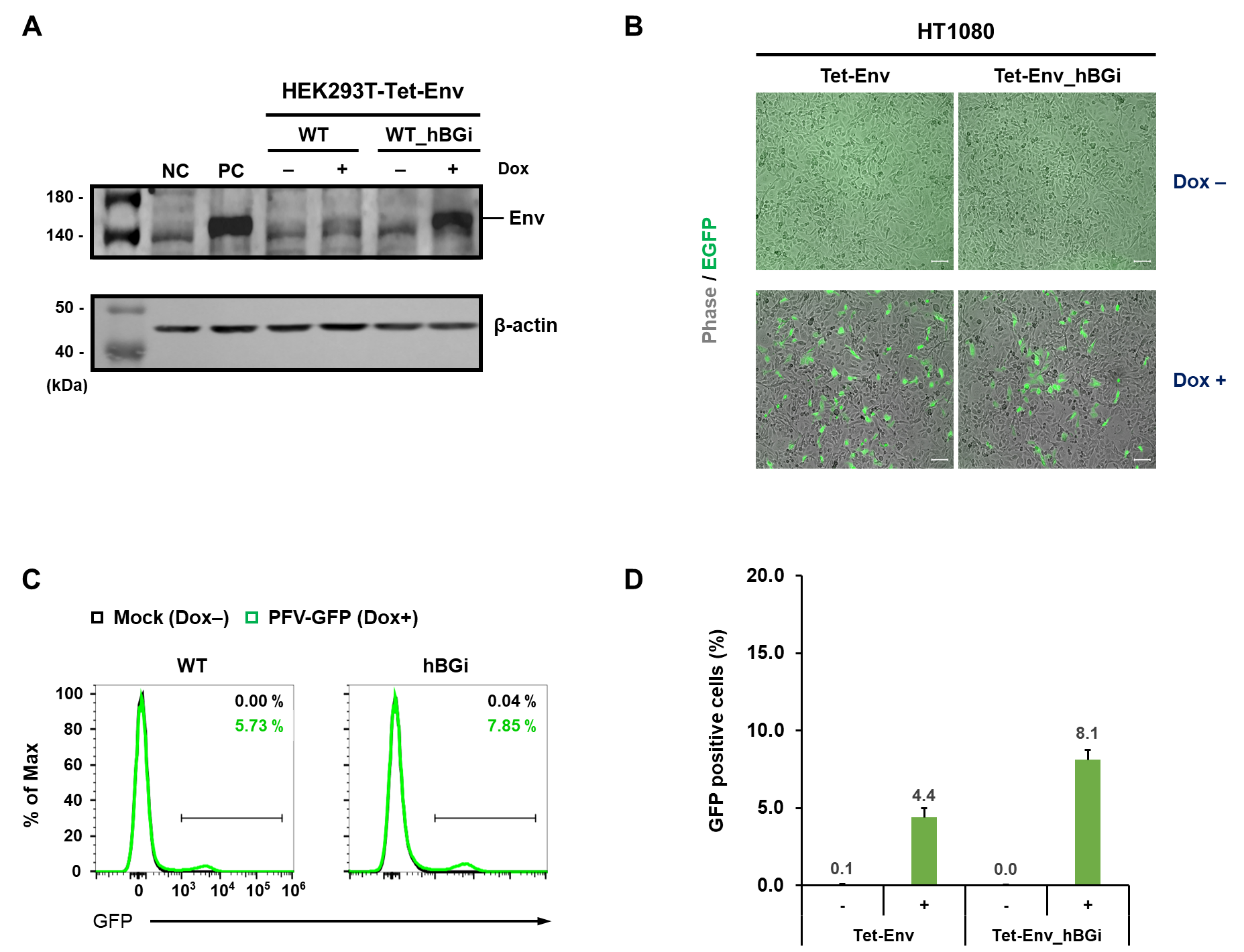
**

**Supplementary Figure 5.** Insertion of a human β-globin intron (hBGi) enhances Tet-On-inducible Env expression. **A**. Western blot analysis confirmed the establishment of HEK293T cells harboring Tet-Env cassettes. Cell lysates were analyzed by western blotting using anti-Env and anti-β-actin antibodies. The letters above the lanes indicate Env type and Dox treatment. **B**. Functional validation using an infection assay. HT1080 cells were infected with viral particles produced from Tet-Env-293T clones following Dox induction. Successful production of infectious viruses was confirmed by GFP expression observed under fluorescence microscopy. **C**. Flow cytometry analysis of HT1080 cells infected with supernatants from individual TetEnv-293T clones with or without Dox treatment. **D**. Quantification of GFP-positive HT1080 cells by flow cytometry, summarizing means ± SEM from biological triplicates.

**Supplementary Figure 6.** Overview of PFV Env variants transduction efficiency normalized to WT. Transduction efficiencies of PFV Env variants were quantified using PFV-EGFP particles and normalized to the mean value of the wild-type Env (WT). The panel includes point mutants (R298A, R440A, E446A), upper-domain (UD) variants, lower-domain (LD) variants, double/triple mutants, and the PFV-SFVgorII residue Env. Each symbol represents an individual biological replicate (n ≥ 3), and bars indicate the mean ± SEM.


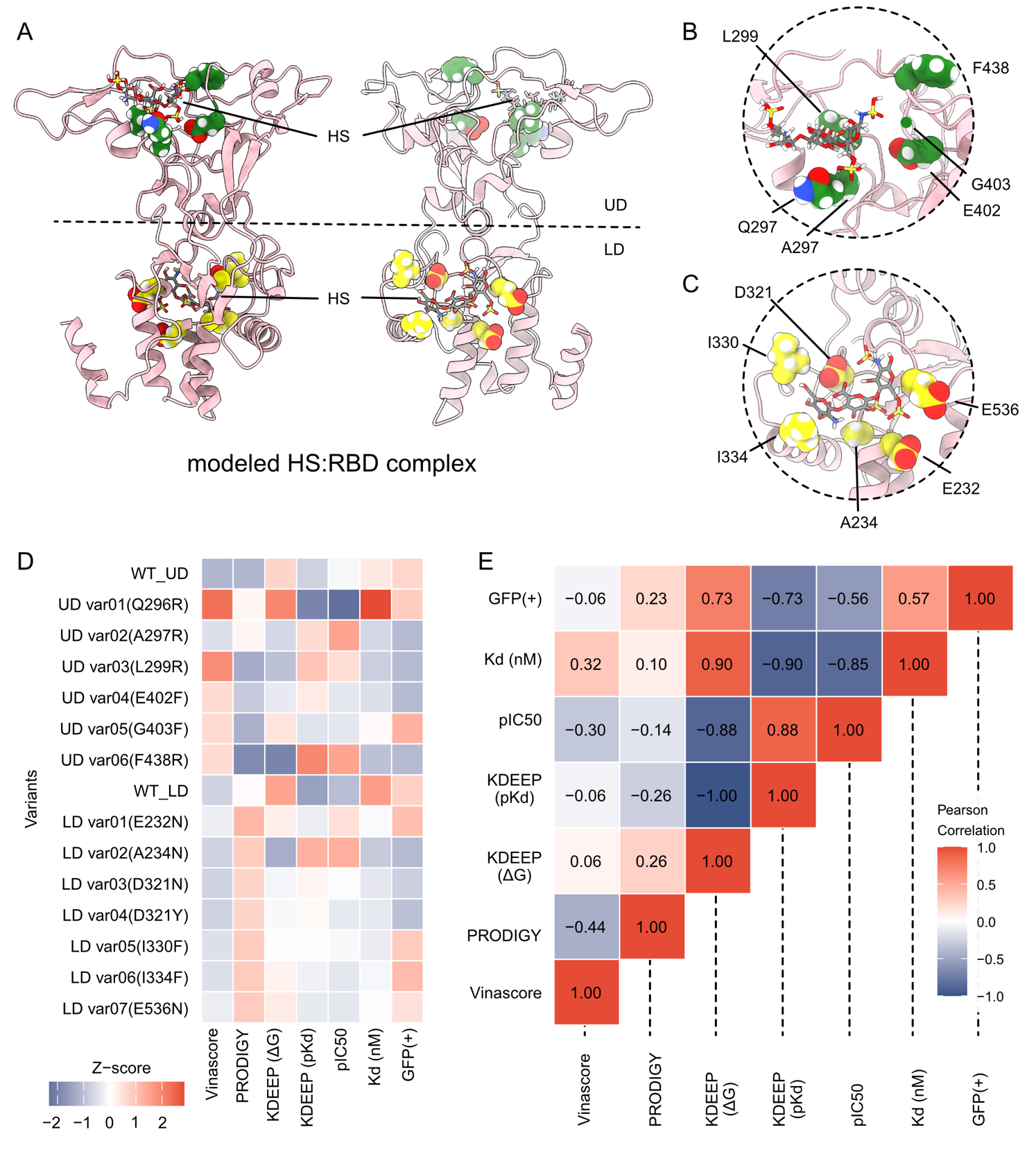


**Supplementary Figure 7.** Structural simulation of variants impacts onto interaction between HS and PFV Env RBD and statistical correlation of in silico and experimental data. **A.** overall structure of PFV Env RBD domain in complex with modeled HS on upper domain (UD) and lower domain (LD). Modeled PFV Env RBD represented as cartoon representation. The heparan sulfate (HS) molecules are modeled as ball and stick model. The variants position in UD and LD are modeled sphere model and colored green and yellow, respectively. **B-C.** Close-up representation of the UD **(B)** and LD **(C)** interaction interface. The residues involved in mutagenesis in current study are labeled. **D.** Heatmap representation of the Z-score distribution for PFV variants across in docking score (vina score), PRODIGY calculated energy (PRODIGY), KDEEP calculated energy (ΔG), predicted Kd (pKd), predicted IC50, calculated Kd, and functional readout (GFP +) parameters. For the affinity parameters including vinascore, PRODIGY, KDEEP, and Kd, the blue color (low Z-score) relevant to high binding affinity. For the experimental transduction read out (GFP +), the red color (high Z-score) stands for the high potential. **E.** Pearson correlation matrix of biophysical and functional parameters. The matrix displays the Pearson correlation coefficient (r) between all tested metrics.


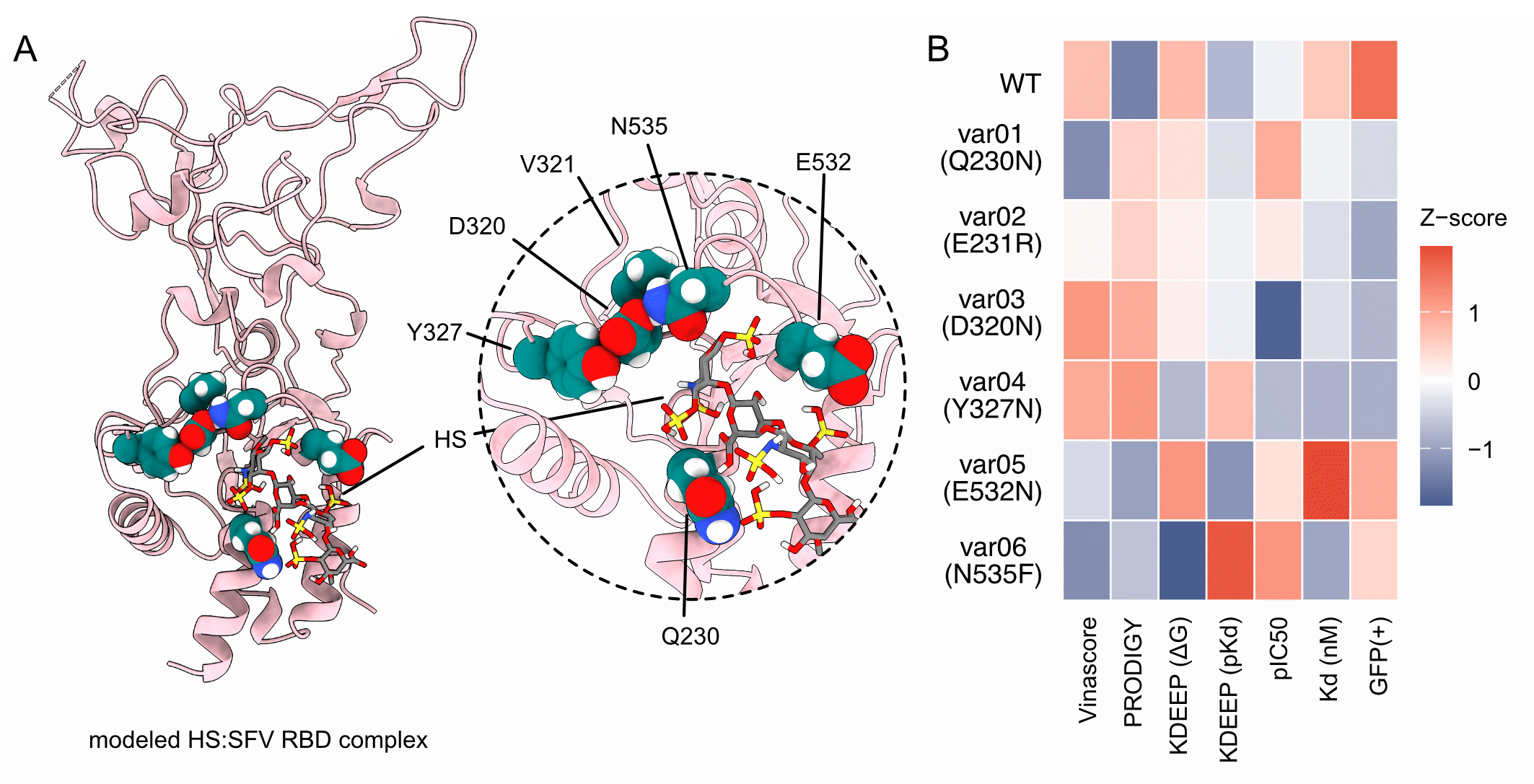
**Supplementary Figure 8.** The structural and spatial position of the chimeric Env variants and predicted binding affinity. **A.** The cartoon representation of the RBD domain of chimeric Env protein in complex with HS. The HS molecules and the variants residues are represented by ball and stick model and sphere, respectively. The zoomed view of the interaction interface are in dotted circle and the residues are labeled. **B.** Heatmap representation of the Z-score distribution for PFV variants across in docking score (vina score), PRODIGY calculated energy (PRODIGY), KDEEP calculated energy (ΔG), predicted Kd (pKd), predicted IC50, calculated Kd, and functional readout (GFP +) parameters. For the affinity parameters including vina score, PRODIGY, KDEEP, and Kd, the blue color (low Z-score) relevant to high binding affinity. For the experimental transduction read out (GFP +), the red color (high Z-score) stands for the high potential.


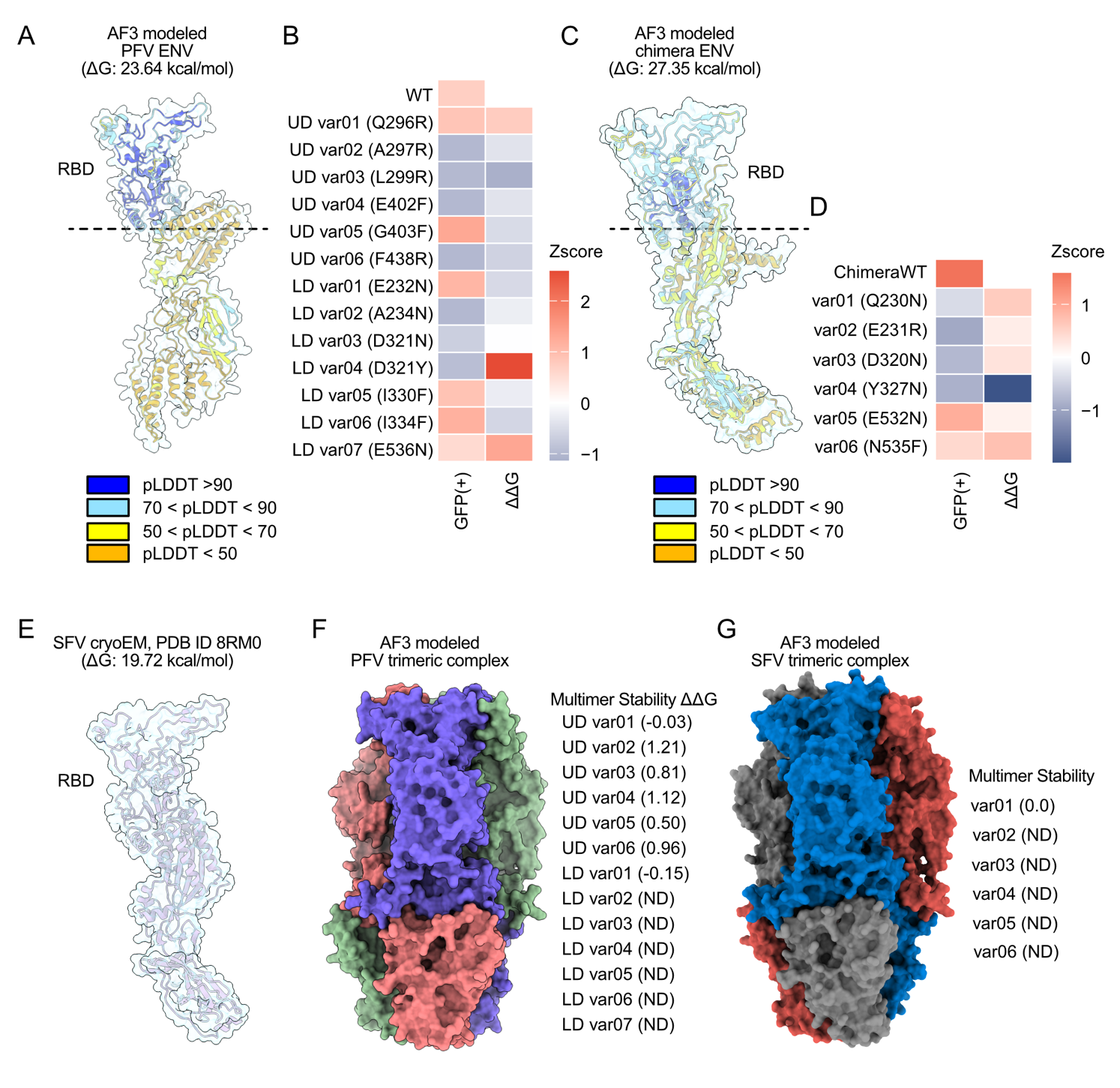


**Supplement Figure 9.** *in silico* validation of structural stability and trimeric assembly for chimeric Env. **A.** AlphaFold3 (AF3) modeled structure of monomeric PFV Env. The structure is colored according to predicted Local Distance Difference Test (pLDDT) confidence scores. The very high (>90), confident (70-90), low (50-70), and very low (<70) were colored blue, skyblue, yellow, and orange, respectively. The global thermodynamic stability (ΔG) is 23.64 labeled. **B.** Heatmaps comparing experimental infectivity (GFP +) with calculated folding stability (ΔΔG) for UD and LD variants of PFV. The low Z-scored ΔΔG stands for stable structure. **C.** AF3 modeled structure of monomeric chimeric Env. The structure is colored according to predicted Local Distance Difference Test (pLDDT) confidence scores. The very high (>90), confident (70-90), low (50-70), and very low (<70) were colored blue, skyblue, yellow, and orange, respectively. The global thermodynamic stability (ΔG) is 27.35 kcal/mol labeled. **D.** Heatmaps comparing experimental infectivity (GFP +) with calculated folding stability (ΔΔG) for UD and LD variants of chimeric Env. **E.** The cryoEM structure of simian foamy virus env protein (SFV) (PDB ID 8RM0) is represented by cartoon and surface. The predicted thermodynamic stability (ΔG) is 19.72 kcal/mol labeled. **F,G**. AF3 modeled trimeric complex of PFV Env and chimeric Env are represented surface model. The calculated trimer stability (ΔΔG) for variants are labeled.

# Supplementary Tables

# Supplementary Table 1. Nucleotide sequences of primers used for site-directed mutagenesis.

| Mutants | Primer sequence (5’->3’) |
| --- | --- |
| R298A | CAG GCC **GCC** CTG GGC TCC TTT TAC |
| R440A | C GCC TGC AGA TTC TGG **GCC** TCC AAA AAC GAA AAG |
| E446A | CC AAA AAC GAA AAG **GCC** GAA ACC AAG TGC C |
| UD var1 (Q296R) | CA AAA AGC AAG TAC GGC **CGG** GCC AGA CTG G |
| UD var2 (A297R) | GC AAG TAC GGC CAG **CGG** AGA CTG G |
| UD var3 (L299R) | GC CAG GCC AGA **CGG** GGC TCC TTT TAC ATC |
| LD var1 (E232N) | C CAC AGA AAG TGT TAC CAA **AAC** TTC GCC AAC TGC TAC CTC |
| LD var2 (A234N) | GA AAG TGT TAC CAA GAG TTC **AAT** AAC TGC TAC CTC GTG AAG |
| LD var3 (D321N) | GC CAC GTG CTG TTT TGC AGC **AAC** CAG CTG TAC AGC AAA TG |
| LD var4 (D321Y) | GC CAC GTG CTG TTT TGC AGC **TAC** CAG CTG TAC AGC AAA TG |
| LD var5 (I330F) | G TAC AGC AAA TGG TAC AAC **TTC** GAA AAC ACC ATC GAG CAG |
| LD var6 (I334F) | G TAC AAC ATC GAA AAC ACC **TTC** GAG CAG AAC GAG CGG TTT C |
| LD var7 (E536N) | C ACC GGC ACC CCT GTG AAC **AAT** ATG CCT AAC GCC AGG GCC |
| LD var5+6 | G TAC AGC AAA TGG TAC AAC **TTC** GAA AAC ACC **TTC** GAG CAG |

Point mutations introduced into the original sequences are indicated by underlining.

**Supplementary** **Table 2.** Summary of transduction efficiency of all Env variants tested.

| # | Plasmid name | Point mutation | Strategy | Transduction efficiency |
| --- | --- | --- | --- | --- |
| 1 | pCMV-Env |  |  | + + + |
| 2 | pCMV-Env R298A | R298A | Chemically neutral | – |
| 3 | pCMV-Env R440A | R440A | Chemically neutral | – |
| 4 | pCMV-Env E446A | E446A | Chemically neutral | – |
| 5 | pCMV-Env UD var1 | Q296R | (+) charged | + + + + |
| 6 | pCMV-Env UD var2 | A297R | (+) charged | – |
| 7 | pCMV-Env UD var3 | L299R | (+) charged | – |
| 8 | pCMV-Env UD var4 | E402F | *π* (pi) stacking | – |
| 9 | **pCMV-Env UD var5** | **G403F** | ***π* (pi) stacking** | **+ + + + + +** |
| 10 | pCMV-Env UD var6 | F438R | (+) charged | – |
| 11 | pCMV-Env SFVgorII | N342K, K343R, A369R | SFVgorII Env HS binding residue | – |
| 12 | **pCMV-Env LD var1** | **E232N** | **Polar** | **+ + + + + +** |
| 13 | pCMV-Env LD var2 | A234N | Polar | – |
| 14 | pCMV-Env LD var3 | D321N | Polar | – |
| 15 | pCMV-Env LD var4 | D321Y | *π* (pi) stacking | – |
| 16 | pCMV-Env LD var5 | I330F | *π* (pi) stacking | **+ + + +** |
| 17 | **pCMV-Env LD var6** | **I334F** | ***π* (pi) stacking** | **+ + + + + +** |
| 18 | pCMV-Env LD var7 | E536N | Polar | + + + |
| 19 | pCMV-Env LD var17 | E232N E536N |  | – |
| 20 | pCMV-Env LD var56 | I330F I334F |  | + + + |
| 21 | **pCMV-Env LD var156** | **E232N I330F I334F** |  | **+ + + + + +** |
| 22 | **pCMV-Env UD var6 LD var56** | **G403F I330F I334F** |  | **+ + + + + + + +** |
| 23 | pCMV-Chimeric Env | RBD replace | Cross Domain | **+ +** |
| 24 | pCMV-Chimeric Env var1 | Q230N | Polar | – |
| 25 | pCMV-Chimeric Env var2 | E231N | (+) charged | – |
| 26 | pCMV-Chimeric Env var3 | D320N | Polar | – |
| 27 | pCMV-Chimeric Env var4 | Y327N | Polar | – |
| 28 | pCMV-Chimeric Env var5 | E532N | Polar | + |
| 29 | pCMV-Chimeric Env var6 | N535F | *π* (pi) stacking | – |

Changes in relative transduction efficiency were represented as follows:

A ″+″ was added for each ≥10% increase, ″+″ was removed for each ≥10% decrease, and a single ″–″ for negligible or low levels.

**Supplementary Table 3**. Comparison of predicted binding affinity of variants and its cellular activity.

| **Binding Domain** | **Mutation** | **PRODIGY score (**ΔG**)*** | **GFP (+) cell (%)** | KDEEP ΔG^#^ | KDEEP pKd | pIC50 | Kd (nM)^$^ |
| --- | --- | --- | --- | --- | --- | --- | --- |
| **UD** | WT | -5.35 | 46.7 | -7.7399 | 5.7332 | 6.2536 | 1848.4 |
|  | UD var01 (Q296R) | -5.19 | 50.7 | -7.1745 | 5.3144 | 5.5002 | 4848.4 |
|  | UD var02 (A297R) | -5.19 | 0.1 | -8.3384 | 6.1766 | 6.8053 | 665.9 |
|  | UD var03 (L299R) | -5.37 | 0.5 | -8.5026 | 6.2983 | 6.4742 | 503.2 |
|  | UD var04 (E402F) | -5.31 | 0.2 | -8.2124 | 6.0833 | 6.1621 | 825.5 |
|  | UD var05 (G403F) | -5.36 | 62.3 | -7.859 | 5.8215 | 6.1624 | 1508.3 |
|  | UD var06 (F438R) | -5.44 | 0.1 | -8.9453 | 6.6262 | 6.798 | 236.5 |
| **LD** | WT | -5.2 | 49.4 | -7.3925 | 5.476 | 5.9351 | 3342 |
|  | LD var01 (E232N) | -5.06 | 56.6 | -7.9399 | 5.8814 | 6.4679 | 1314 |
|  | LD var02 (A234N) | -5.1 | 0.1 | -8.6322 | 6.3942 | 6.7372 | 403.5 |
|  | LD var03 (D321N) | -5.12 | 9.0 | -8.129 | 6.0215 | 6.271 | 951.7 |
|  | LD var04 (D321Y) | -5.12 | 2.39 | -8.1015 | 6.0011 | 6.1772 | 997.5 |
|  | LD var05 (I330F) | -5.1 | 51.4 | -8.0694 | 5.9773 | 6.2557 | 1053.7 |
|  | LD var06 (I334F) | -5.1 | 58.5 | -7.9661 | 5.9008 | 6.1619 | 1256.6 |
|  | LD var07 (E536N) | -5.1 | 41.9 | -7.9152 | 5.8631 | 6.163 | 1370.6 |
| **sFV (chimera)** | WT | -5.43 | 56.6 | -10.2122 | 7.5646 | 6.5583 | 27.2521 |
|  | sFV var01 (Q230N) | -5.18 | 13.3 | -10.5774 | 7.8351 | 7.261 | 14.618 |
|  | sFV var02 (E231R) | -5.18 | 1.14 | -10.7334 | 7.9507 | 6.7957 | 11.202 |
|  | sFV var03 (D320N) | -5.12 | 5.00 | -10.7184 | 7.9396 | 5.462 | 11.4921 |
|  | sFV var04 (Y327N) | -5.09 | 3.19 | -11.4905 | 8.5115 | 6.1594 | 3.079 |
|  | sFV var05 (E532N) | -5.38 | 42.4 | -9.8477 | 7.2946 | 6.868 | 50.745 |
|  | sFV var06 (N535F) | -5.33 | 31.6 | -12.4464 | 9.2196 | 7.4438 | 0.603 |

*The binding free energy (ΔG, kcal/mol) calculated by using PRODIGY server (Vangone et al., 2019) after docking with AF3 modeled variants (Abramson et al., 2024).

#The affinity score (ΔG, kcal/mol) calculated by using KDEEP, a state-of-art deep learning based affinity calculation software (Jimenez et al., 2018).

$The converted Kd calculated from the converting pKd from KDEEP software under equation expressed as Kd values (-log10pKd) based on the thermodynamic relationship ΔG = RTln(Kd).

**Reference**

Abramson, J., Adler, J., Dunger, J., Evans, R., Green, T., Pritzel, A., et al. (2024). Accurate structure prediction of biomolecular interactions with AlphaFold 3. *Nature* 630(8016)**,** 493-500. doi: 10.1038/s41586-024-07487-w.

Fernandez, I., Dynesen, L.T., Coquin, Y., Pederzoli, R., Brun, D., Haouz, A., et al. (2023). The crystal structure of a simian Foamy Virus receptor binding domain provides clues about entry into host cells. *Nat Commun* 14(1)**,** 1262. doi: 10.1038/s41467-023-36923-0.

Jimenez, J., Skalic, M., Martinez-Rosell, G., and De Fabritiis, G. (2018). K(DEEP): Protein-Ligand Absolute Binding Affinity Prediction via 3D-Convolutional Neural Networks. *J Chem Inf Model* 58(2)**,** 287-296. doi: 10.1021/acs.jcim.7b00650.

Vangone, A., Schaarschmidt, J., Koukos, P., Geng, C., Citro, N., Trellet, M.E., et al. (2019). Large-scale prediction of binding affinity in protein-small ligand complexes: the PRODIGY-LIG web server. *Bioinformatics* 35(9)**,** 1585-1587. doi: 10.1093/bioinformatics/bty816.
